# Supplementary material for: A Multi-Stage Approach for Cardiovascular Risk Assessment from Retinal Images Using an Amalgamation of Deep Learning and Computer Vision Techniques
Source: Diagnostics (Basel). 2024 Apr 29;14(9):928. doi: 10.3390/diagnostics14090928 (PMC11083022; doi:10.3390/diagnostics14090928)
Supplement: Supplementary file 1 [file diagnostics-14-00928-s001.zip › diagnostics-2947642-supplementary.pdf]

A Multi-stage Approach for Cardiovascular Risk Assessment from Retinal Images using an Amalgamation of Deep Learning and Computer Vision Techniques

Deepthi K Prasad [a], Madhura Prakash M [a], Meghna S Kulkarni [a], Spoorthi K [a], Venkatakrishnan S [a], Madhulika Chakravarthi [b] and Anusha Ramesh [c]

[a] Research and Development, Image Processing and Analysis, Forus Health Private Ltd, Bangalore, India

[b] Senior resident, Department of Cardiology, Apollo Hospitals, Bangalore, India

[c] Senior resident, Department of OBGyn, St. Johns Medical College, Bangalore, India

Supplementary Material

Table S1. Pre-processing Algorithm-1 for Binary Cardio Risk Classification Model

**Input Parameter:**<br>The function takes an input image (img_input) as its parameter<br>**Split Channels:**<br>Split the input image into its three-color channels: Blue (B), Green (G), and Red (R)<br>CLAHE (Contrast Limited Adaptive Histogram Equalization) on Green Channel:<br>Create a CLAHE object with a specified clip limit of 1.0 and tile grid size of (25,25)<br>Apply CLAHE to the Green channel (G)<br>**Intensity Scaling on Green Channel:**<br>Multiply the CLAHE-enhanced green channel (C_G) by a scaling factor of 0.8. This step adjusts the intensity.<br>**Merge Channels:**<br>Merge the original blue channel (B), the updated green channel (updated_G), and the original red channel (R) to form the new BGR image<br>**Return Result:**<br>Return the final BGR image (img_new_bgr), where the green channel has undergone CLAHE and intensity scaling

Table S2. Masking and Cropping Algorithm

Input Parameters:
The function takes an image (img), a mask (mask_in), and a tolerance parameter (tol) as inputs
Gray Scale Check:
If the image is 2-dimensional (gray scale), create a binary mask where pixel values are greater than the specified tolerance (tol).
Return the cropped image and corresponding mask using the computed binary mask.
Color Image Processing:
If the image is 3-dimensional (color), convert the image to grayscale Create a binary mask based on the grayscale image where pixel values are greater than the specified tolerance (tol)
Check Cropped Image Shape:
Check the shape of the first channel of the original image after applying the binary mask
If the resulting shape is zero, the image is too dark, so return the original image and mask.
Extract Channels and Stack:
If the image is not too dark, extract each channel separately based on the binary mask.
Stack the three channels together to form the cropped color image.
Handling Mask Channels:
Try to extract each channel from the input mask (mask_in) based on the binary mask.
If successful, stack the three mask channels together. If unsuccessful (due to different sizes), use the original mask.
Additional Handling for Shape Mismatch:
Check if the width dimension of the original image (shape) is not equal to the width dimension of the input mask (mask_in.shape[1]). If unequal, use the original mask (mask_in)
Result:
Return the final cropped color image (img) and the corresponding mask (msk)

Table S3. Pre-Processing Algorithm for Vessel Enhancement

Input Parameter:
The function takes an Image as input.
Gamma Adjustment:
Define a nested function adjust_gamma(image, gamma) to perform gamma adjustment on the input image.
Calculate invGamma as the inverse of the specified gamma value.
Create a lookup table using NumPy to adjust pixel values in the range [0, 255].
Apply the lookup table using OpenCV's LUT (Look-Up Table) function to adjust the gamma of the image.
Image Conversion and CLAHE:
Assign the input image to the variable img.
Create a Contrast Limited Adaptive Histogram Equalization (CLAHE) object using cv2.createCLAHE with specified parameters (clipLimit=2.5 and tileGridSize= (15,15))
Split Channels:

Extract three color channels (C1, C2, C3) from the image array.
CLAHE Application:
Apply CLAHE separately to the second channel (C2) using clahe.apply and store the result in CH1.
Merge Channels:
Merge the modified second channel (CH1) with the other two channels to form a new image (AA)
Gamma Adjustment on Merged Image:
Apply the previously defined gamma adjustment function (adjust_gamma) to the merged image (AA) with a specified gamma value of 2.5.
Convert to Grayscale:
Convert the gamma-adjusted image (gi) to grayscale
Merge Grayscale Channels:
Merge the grayscale image (gi) with itself to form a 3-channel grayscale image (Gi)
Return Result:
Return the final processed image (Gi) as the output of the function.

```
 Layer (type)                Output Shape              Param #
=================================================================
 input_2 (InputLayer)        [(None, 224, 224, 3)]     0

 batch_normalization (BatchN  (None, 224, 224, 3)      12
 ormalization)

 efficientnetb0 (Functional)  (None, 7, 7, 1280)       4049571

 conv2d (Conv2D)             (None, 7, 7, 128)         163968

 flatten (Flatten)           (None, 6272)              0

 dropout (Dropout)           (None, 6272)              0

 dense (Dense)               (None, 512)               3211776

 dropout_1 (Dropout)         (None, 512)               0

 dense_1 (Dense)             (None, 1)                 513

=================================================================
Total params: 7,425,840
Trainable params: 7,383,811
Non-trainable params: 42,029
```

Figure S1. Binary Cardio Risk Classification Model Summary
